# Search for Early Pancreatic Cancer Blood Biomarkers in Five European Prospective Population Biobanks Using Metabolomics

**DOI:** 10.1210/en.2019-00165

**Published:** 2019-05-24

**Authors:** Jesse Fest, Lisanne S Vijfhuizen, Jelle J Goeman, Olga Veth, Anni Joensuu, Markus Perola, Satu Männistö, Eivind Ness-Jensen, Kristian Hveem, Toomas Haller, Neeme Tonisson, Kairit Mikkel, Andres Metspalu, Cornelia M van Duijn, Arfan Ikram, Bruno H Stricker, Rikje Ruiter, Casper H J van Eijck, Gert-Jan B van Ommen, Peter A C ʼt Hoen

**Affiliations:** 1 Department of Surgery, Erasmus Medical Center, Rotterdam, Netherlands; 2 Department of Epidemiology, Erasmus Medical Center, Rotterdam, Netherlands; 3 Department of Human Genetics, Leiden University Medical Center, Leiden, Netherlands; 4 Department of Biomedical Data Sciences, Leiden University Medical Center, Leiden, Netherlands; 5 Department of Public Health Solutions, National Institute for Health and Welfare, Helsinki, Finland; 6 Research Program for Clinical and Molecular Metabolism, Faculty of Medicine, University of Helsinki, Helsinki, Finland; 7 HUNT Research Center, Department of Public Health and Nursing, Norwegian University of Science and Technology, Levanger, Norway; 8 Institute of Genomics, University of Tartu, Tartu, Estonia; 9 Department of Clinical Genetics, Tartu University Hospital, Tartu, Estonia; 10 Center for Molecular and Biomolecular Informatics, Radboud Institute for Molecular Life Sciences, Radboud University Medical Center, Nijmegen, Netherlands

## Abstract

Most patients with pancreatic cancer present with advanced disease and die within the first year after diagnosis. Predictive biomarkers that signal the presence of pancreatic cancer in an early stage are desperately needed. We aimed to identify new and validate previously found plasma metabolomic biomarkers associated with early stages of pancreatic cancer. Prediagnostic blood samples from individuals who were to receive a diagnosis of pancreatic cancer between 1 month and 17 years after sampling (N = 356) and age- and sex-matched controls (N = 887) were collected from five large population cohorts (HUNT2, HUNT3, FINRISK, Estonian Biobank, Rotterdam Study). We applied proton nuclear magnetic resonance–based metabolomics on the Nightingale platform. Logistic regression identified two interesting hits: glutamine (*P* = 0.011) and histidine (*P* = 0.012), with Westfall–Young family-wise error rate adjusted *P* values of 0.43 for both. Stratification in quintiles showed a 1.5-fold elevated risk for the lowest 20% of glutamine and a 2.2-fold increased risk for the lowest 20% of histidine. Stratification by time to diagnosis suggested glutamine to be involved in an earlier process (2 to 5 years before diagnosis), and histidine in a process closer to the actual onset (<2 years). Our data did not support the branched-chain amino acids identified earlier in several US cohorts as potential biomarkers for pancreatic cancer. Thus, although we identified glutamine and histidine as potential biomarkers of biological interest, our results imply that a study at this scale does not yield metabolomic biomarkers with sufficient predictive value to be clinically useful *per se* as prognostic biomarkers.

Pancreatic cancer is one of the most lethal cancers worldwide and is increasingly common (1–3). Most patients present with advanced and thus incurable disease and die within a year of the initial diagnosis (3, 4). There is an imminent need to identify these patients earlier in the disease process, as patients with resectable, nonmetastatic cancer can potentially be cured. For many cancers it takes several years for a local malignant lesion to progress to fully metastasized disease, and pancreatic cancer is no exception (5). Thus, there should be a window of opportunity for timely detection and intervention. Unfortunately, for early, presymptomatic pancreatic cancer currently no specific biomarkers are available. The identification of predictive biomarkers is complicated by the low incidence rate of the disease, estimated at 7 to 12 cases per 100,000 adult person years in the Western European population (6, 7).

It is well known that the development and progression of pancreatic cancer are associated with alterations in systemic metabolism. Patients may present with glucose intolerance, anorexia, and severe weight loss (3, 8). In line with this, circulating metabolites have been proposed as a potentially useful screening tool in pancreatic cancer (9–16). The study by Mayers *et al.* (11) stood out from other metabolomic biomarker studies, as they analyzed blood samples taken 2 to more >10 years prior to diagnosis. They found an elevation of circulating branched-chain amino acids as an early event in the development of pancreatic cancer (11).

Considering these metabolomics biomarkers as promising, we set out to replicate these findings independently in five large European population cohorts and find additional biomarkers associated with early stages of pancreatic cancer, using a different platform, proton nuclear magnetic resonance (^1^H-NMR) instead of liquid chromatography followed by mass spectrometry. This is a retrospective study where biobanked samples from population cohorts were cross-checked with the national cancer registries to identify samples from individuals who were diagnosed with pancreatic cancer after blood sampling. This was done because truly prospective studies are almost infeasible with low-incident diseases such as pancreatic cancer.

## Methods

### Study population

Our study population consisted of pancreatic cancer cases and controls, drawn from five national European cohorts, collaborating in the Biobanking and Biomolecular Resources Research Infrastructure Large Population Cohorts (BBMRI-LPC; www.bbmri-lpc-biobanks.eu) and the cross-infrastructure project CORBEL (www.corbel-project.eu): the Estonian Genome Center of the University of Tartu study (EGCUT), the FINRISK Study (FR), the Nord-Trøndelag Health Study (HUNT2 and HUNT3), and the Rotterdam Study (RS).

EGCUT is a volunteer-based sample of the Estonian resident adult population aged ≥18 years, started in 1999, and currently has nearly 152,000 participants (17). EGCUT can link its own database with the national electronic databases (eight total) to constantly update the phenotype information for the subjects. Every entry in the biobank consists of: (i) biological samples, (ii) answers to the questions of a computer-assisted personal interview conducted at the doctor’s office, (iii) objective measurements performed at the doctor’s office, (iv) electronic health data from various databases, (v) genotype data from array genotyping, exome sequencing, or whole-genome sequencing, and (vi) biomedical data obtained by performing various assays on the material collected.

FINRISK was initiated in 1972 and includes a collection of cross-sectional surveys in the adult (25- to 74-year-old) permanent residents of selected geographical areas of Finland. Altogether, FINRISK had nine cross-sectional surveys performed every fifth year by the National Institute for Health and Welfare, including a total of 101,451 invitees (18). Participants in this study were selected from the FINRISK 1997, 2002, and 2007 surveys. There are no reexaminations except for occasional people who were selected to more than one independent survey by chance. Follow-up is carried out through record linkages to national administrative registers (such as the Causes of Death Register and Cancer Register) by using a unique personal identity code (19).

HUNT includes repeated surveys of a large population-based cohort in Norway. Data from 116,044 individuals aged ≥20 years from HUNT2 (1995 to 1997, n = 65,237) and HUNT3 (2006 to 2008, n = 50,807) were used in this study. Individuals who participated in both HUNT2 and HUNT3 were only included in the current study as part of HUNT3. Similar to FINRISK, follow-up is carried out through record linkages to national administrative registers (such as the Causes of Death Register and Cancer Register) by using a unique personal identity code (20).

The RS is an ongoing, population-based cohort study in a suburban area of Rotterdam, Netherlands. At baseline, all participants underwent both an interview at home and an extensive set of examinations at a research facility, and blood samples (both plasma and serum) were collected. At each follow-up point, blood samples were collected. It was initiated in 1989 and has enrolled 14,926 individuals of ≥45 years of age since then. Follow-up is carried out every three to four years. An automated follow-up system is linked to digital medical records from general practitioners (including discharge letters from hospitals) and linked to a registry of histopathology and cytopathology [Pathologisch-Anatomisch Landelijk Geautomatiseerd Archief (PALGA)] and to Landelijke Medische Registratie (LMR) and the Integraal Kankercentrum Nederland (IKNL) (21, 22).

All participants of the respective cohorts provided written informed consent. The current study was approved by the local ethics committee of each study.

### Selection of cases and controls

We included incident pancreatic cancer cases, confirmed by pathology and diagnosed after blood collection. Cases were identified through national cancer registries and through independent review of medical records. For diagnosis of pancreatic cancer, we used the ICD-10 C25.0 code. Deaths were ascertained through the national registries. We excluded cases that lived >5 years after diagnosis to avoid false-positive diagnoses (23–25).

For each case, we selected two (in RS one, in EGCUT four) random controls, matching on cohort, sex, age at sample collection (±2 years), and time of blood collection. Controls were those who were alive and without a diagnosis of pancreatic cancer at time of the case’s diagnosis date.

### Ascertainment of other covariates

The following covariate data were obtained from questionnaires and physical examination before blood collection: body mass index (BMI; kg/m^2^), smoking status (current/former/never), type 2 diabetes mellitus (T2DM) status, and fasting status (<4 hours/4 to 8 hours/>8 hours).

### Metabolite profiling and quality control

Serum was collected from serum separator tubes with glass or silica clot activators, with or without gel as separator, and stored at −80°C. EDTA plasma was collected from Vacutainer tubes and processed and stored at −80°C within 48 hours of blood draw. Metabolites were quantified from EDTA plasma (EGCUT) or serum (HUNT2, HUNT3, FR, RS) samples using a high-throughput ^1^H-NMR metabolomics platform (Nightingale Health, Helsinki, Finland; https://nightingalehealth.com/). This platform provides simultaneous quantification of 147 individual metabolites and 79 metabolite ratios, for example, routine lipids, lipoprotein subclass profiling with lipid concentrations within 14 subclasses, esterified fatty acid composition, and various low‐molecular-weight metabolites, including amino acids, ketone bodies, and gluconeogenesis‐related metabolites in molar concentration units. Details of the experimentation and applications of the platform have been described previously (26).

Metabolite measures that failed quality control (in particular for glutamine, pyruvate, glycerol, hydroxybutyrate, and acetate) were excluded from the analysis on a per-individual basis. One metabolite measure (glycerol) with >10% missing values was excluded entirely, resulting in a final number of 146 metabolite measures and 79 ratios. Outliers (>5 SD) were removed in concordance with previous research in this field (27).

### Statistical analysis

Differences in baseline characteristics between cases and controls were assessed for each cohort separately using two-tailed Student *t* tests (continuous variables) or *χ*^2^ tests (categorical variables).

Metabolite measurements were raised by 1 to allow log transformation. Thereafter, all metabolite values were log-transformed and scaled to obtain unit SD for each cohort. They were included as continuous variables in logistic regression models and adjusted for matching factors (sex and age at sample collection, minimally adjusted model). In our main model on the pooled data from all of the cohorts, we additionally adjusted for BMI, smoking status, T2DM status, fasting status, and cohort. *P* values were corrected for multiple testing using Westfall and Young family-wise error rate, an appropriate method given the strong correlations between the measurements of the different metabolites (28). To provide estimates of effect magnitude, significant metabolites were again examined in logistic regression models after categorization in quintiles. Quintiles were generated based on the metabolite values in controls only. Results are presented as ORs and 95% CIs.

As an alternative for the pooling of the data from the different cohorts, we also performed a logistic regression per cohort (with sex, age, BMI, smoking status, T2DM, and fasting status as covariates) and a subsequent meta-analysis. The obtained estimates for the metabolite measures and their standard errors were used in a random effects meta-analysis using the R package meta 4.9.2 (29). A random effects model was chosen to account for possible heterogeneity due to differences in disease assessment, sample processing, and sample collection between cohorts. Heterogeneity was assessed using the *I*^2^ statistic and by visual inspection of forest plots. *P* values from the meta-analysis were corrected for multiple-testing using a Bonferroni–Holm test.

### LASSO regression to evaluate additive effect of metabolomics biomarkers on top of clinical predictors

To select biomarkers with predictive value, we applied a fivefold cross-validated penalized least absolute shrinkage and selection operator (LASSO) regression with the penalized package version 0.9-51 (30). The clinical covariates (sex, age, BMI, smoking status, T2DM status, fasting status, and cohort) were not penalized and thus were always present in the model. We performed a stratified analysis, including all controls but only cases who developed pancreatic cancer within 2 years or within 5 years after blood sampling or including all cases. For the variable selection, the data were split randomly into a data set for variable selection (70% of the data, with 35% for training and 35% for cross-validation) and a data set for performance testing (30% of the data). We compared the performance of the null model (with only the clinical covariates) with the model that included the selected metabolites using an ordinary least squares regression model. The performance of the different model was assessed by evaluating the area under the receiver operator curve (AUC).

### General

Analyses were performed using the software packages meta 4.9-2, Penalized 0.9-51, Globaltest 5.24.0, InformationValue 1.2.3, ROCR 1.0-7, RColorBrewer 1.1-2, and ggplot2 3.0.0 for R version 3.2.3. All scripts are available in an online repository (31).

### Availability of data

Additional files with complete results are available in an online repository (32). For reasons of privacy protection, raw data are only available upon request.

## Results

### Study population and measurements

**Figure 1.**
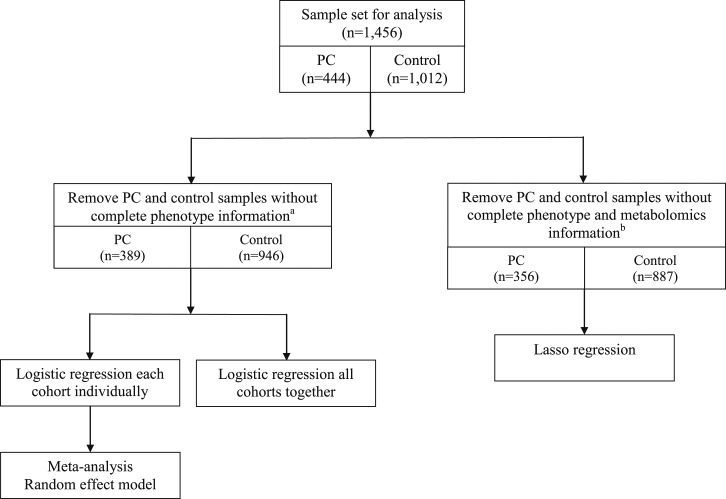
Schematic overview of the sample set used for data analysis and the different data analysis approaches performed in the current study. ^a^Any individual containing missing values in metabolomics measurements or phenotypical information were assumed to be missing at random and were removed from the data set. ^b^Any individual containing missing values in phenotypical information were removed from the data set. PC, pancreatic cancer.

Cross-checking of the individuals in the five population cohorts included in this study with the national cancer registries enabled us to identify 444 prediagnostic samples from subjects who received diagnosis of pancreatic cancer between 1 month and 17 years after blood sampling (median, 4.68 years). We subsequently selected 1012 sex- and age-matched controls from the same cohorts (Fig. 1). Baseline characteristics for all cohorts are shown in Table 1. Baseline characteristics differed significantly between cohorts (in particular for sex, BMI, T2DM, and fasting status). Cases were significantly and consistently enriched for T2DM patients and smokers, in line with the comorbidity of pancreatic cancer and T2DM and smoking as a known risk factors for pancreatic cancer (Table 1). We reliably quantified 146 blood metabolites and 79 metabolite ratios. Figure 1 shows the number of participants remaining after quality control and after assessment of the completeness of phenotype information in the different analyses performed.

**Table 1.** Baseline Characteristics

	HUNT2 (n = 590)			HUNT3 (n = 194)			EGCUT (n = 227)			FR (n = 272)			RS (n = 173)			ALL (n = 1456)		
	Cases	Controls	Statistics	Cases	Controls	Statistics	Cases	Controls	Statistics	Cases	Controls	Statistics	Cases	Controls	Statistics	Cases	Controls	Statistics
Total, n (%)	158 (26.8%)	432 (73.2%)		64 (33%)	130 (67%)		76 (33.5%)	151 (66.5%)		57 (21%)	215 (79%)		89 (51.4%)	84 (48.6%)		444 (30.5%)	1012 (69.5%)	
Female, n (%)	80 (50.6%)	220 (50.9%)	*P* = 0.95	37 (57.8%)	76 (58.5%)	*P* = 0.93	44 (57.9%)	88 (58.3%)	*P* = 0.96	22 (38.6%)	82 (38.1%)	*P* = 0.95	54 (60.7%)	45 (53.6%)	*P* = 0.35	237 (53.4%)	511 (50.5%)	*P* = 0.31
Age, y, mean (SD)	66.8 ± 11.1	64.8 ± 11.2	*P* = 0.065	69.5 ± 9.5	69.5 ± 9.3	*P* = 0.97	63.4 ± 10.6	63.3 ± 10.5	*P* = 0.94	59.7 ± 8.8	59.8 ± 8.9	*P* = 0.96	71.6 ± 8.5	70.9 ± 9.2	*P* = 0.62	66.6 ± 10.7	64.6 ± 10.8	*P* = 0.001^*a*^
BMI, kg/m^2^, mean (SD)	27.3 ± 4.1	26.9 ± 3.7	*P* = 0.28	28.3 ± 3.6	27 ± 4.2	*P* = 0.025^*a*^	29.1 ± 5.8	28.5 ± 5.4	*P* = 0.40	27.2 ± 3.9	27.7 ± 4.3	*P* = 0.45	27.4 ± 4.1	27.3 ± 4.1	*P* = 0.89	27.8 ± 4.4	27.3 ± 4.3	*P* = 0.078
DM, n (%)	11 (7%)	18 (4.2%)	*P* = 0.16	10 (15.6%)	8 (6.2%)	*P* = 0.034^*a*^	33 (43.4%)	32 (21.2%)	*P* = 0.00059^*a*^	6 (10.5%)	21 (9.8%)	*P* = 0.86	12 (13.5%)	2 (2.4%)	*P* = 0.0074^*a*^	72 (16.2%)	81 (8%)	*P* = 2.77 × 10^−6*a*^
Smoking, n (%)	0 = 49 (31%)	0 = 187 (43.3%)	*P* = 0.00041^*a*^	0 = 22 (34.4%)	0 = 58 (44.6%)	*P* = 0.00077^*a*^	0 = 38 (50%)	0 = 100 (66.2%)	*P* = 0.044^*a*^	0 = 21 (36.8%)	0 = 106 (49.3%)	*P* = 0.043^*a*^	0 = 22 (24.7%)	0 = 20 (23.8%)	*P* = 0.021^*a*^	0 = 152 (34.2%)	0 = 471 (46.5%)	*P* = 8.12 × 10^−9*a*^
	1 = 48 (30.4%)	1 = 140 (32.4%)		1 = 19 (29.7%)	1 = 55 (42.3%)		1 = 18 (23.7%)	1 = 20 (13.2%)		1 = 14 (24.6%)	1 = 66 (30.7%)		1 = 37 (41.6%)	1 = 50 (59.5%)		1 = 136 (30.6%)	1 = 331 (32.7%)	
	2 = 59 (37.3%)	2 = 93 (21.5%)		2 = 20 (31.3%)	2 = 13 (10%)		2 = 20 (26.3%)	2 = 31 (20.5%)		2 = 19 (33.3%)	2 = 41 (19.1%)		2 = 27 (30.3%)	2 = 12 (14.3%)		2 = 145 (32.7%)	2 = 190 (18.8%)	
	NA = 2 (1.3%)	NA = 12 (2.8%)		NA = 3 (4.7%)	NA = 4 (3.1%)		NA = 0 (0%)	NA = 0 (0%)		NA = 3 (5.3%)	NA = 2 (0.9%)		NA = 3 (3.4%)	NA = 2 (2.4%)		NA = 11 (2.5%)	NA = 20 (2%)	
Fasted, n (%)	0 = 134 (84.9%)	0 = 363 (84%)	*P* = 0.95	0 = 46 (71.9%)	0 = 97 (74.6%)	*P* = 0.32	0 = 35 (46.1%)	0 = 97 (64.2%)	*P* = 0.87	0 = 4 (7%)	0 = 4 (1.9%)	*P* = 0.052	0 = 26 (29.2%)	0 = 21 (25%)	*P* = 0.50	0 = 245 (55.2%)	0 = 582 (57.5%)	*P* = 1.49 × 10^−5*a*^
0 = ≤4 h, 1 = 4 h to 8 h	1 = 19 (12%)	1 = 56 (13%)		1 = 9 (14.1%)	1 = 18 (13.8%)		1 = 7 (9.2%)	1 = 24 (15.9%)		1 = 38 (66.7%)	1 = 171 (79.5%)		1 = 1 (1.1%)	1 = 0 (0%)		1 = 74 (16.7%)	1 = 269 (26.6%)	
2 = >8 h	2 = 3 (1.9%)	2 = 9 (2.1%)		2 = 6 (9.4%)	2 = 5 (3.8%)		2 = 6 (7.9%)	2 = 15 (9.9%)		2 = 14 (24.6%)	2 = 40 (18.6%)		2 = 56 (62.9%)	2 = 57 (67.8%)		2 = 85 (19.1%)	2 = 126 (12.5%)	
	NA = 2 (1.3%)	NA = 4 (0.9%)		NA= 3 (4.7%)	NA = 10 (7.7%)		NA = 28 (36.8%)	NA = 15 (9.9%)		NA = 1 (1.8%)	NA = 0 (0%)		NA = 6 (6.7%)	NA = 6 (7.1%)		NA = 40 (9%)	NA = 35 (3.5%)	

Values are number counts (percentages) or mean ± SD. *P* values are from *χ*^2^ test (categorical variables) or Student *t* test (continuous variables) comparing cases and controls.

Abbreviation: NA, not available.

^a^ *P* < 0.05.

### Single-metabolite logistic regression

**Figure 2.**
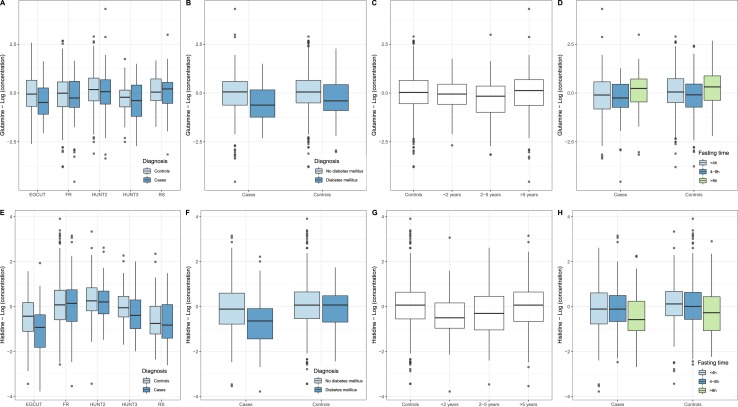
Concentrations (logarithmic scale) of (A–D) glutamine and (E–H) histidine in the blood circulation in controls and cases, that is, those individuals who developed pancreatic cancer within a time window after blood sampling. (A and E) Distribution of the concentrations of controls (light blue) and cases (dark blue) in the different cohorts analyzed (EGCUT, FR, HUNT2, HUNT3, RS). (B and F) Distribution of concentrations in nondiabetics (light blue) and individuals diagnosed with T2DM (dark blue). (C and G) Distribution of concentrations in controls and cases sampled within 2 y before diagnosis, between 2 and 5 y before diagnosis, and >5 y before diagnosis. (D and H) Distribution of concentrations in nonfasting individuals (light blue), individuals who had a meal between 4 and 8 h before blood draw (dark blue), and fasting individuals (green, last meal was >8 h before blood draw). Box plots reflect the distribution of the concentrations in individual samples, including the middle quartiles (25th to 75th percentile of the data points are in the boxes); the horizontal band; the median value; the lower whiskers representing the data points up to 1.5 × the interquartile range (IQR) below the 25th percentile; the upper whiskers representing the data points up to 1.5 × IQR above the 75th percentile; the data points outside these ranges plotted as individual data points.

To identify metabolite biomarkers potentially associated with future pancreatic cancer diagnosis, we performed a separate logistic regression for each metabolite measured. In our primary model, we adjusted for the following covariates: sex, age, BMI, smoking status, T2DM status, fasting status, and cohort. The results of our top metabolites are presented in Table 2. Full data are provided in an online repository (32). Two metabolites demonstrated lower blood levels in cases than in controls and nominal significance: glutamine (*P* = 0.012) and histidine (*P* = 0.011). They were not significant after adjustment for multiple testing (Westfall–Young family-wise error rate adjusted *P* value of 0.43 for both metabolites). To estimate the clinical relevance of our findings, the ORs for being diagnosed with pancreatic cancer within the follow-up time was calculated for an individual with metabolite levels of 1 SD below the mean: these ORs amounted to 1.42 and 1.45 for glutamine and histidine, respectively (see footnote to Table 2). A closer inspection of the levels of glutamine and histidine revealed that the differences were consistently observed across cohorts (Fig. 2A and 2E), except for glutamine in RS and histidine in FR. Glutamine levels were lower in both nondiabetics and patients with diabetes, whereas lower histidine levels were mainly observed in patients with pancreatic cancer who were also diagnosed with T2DM (Fig. 2B). Histidine levels were lower in individuals who developed pancreatic cancer within 2 years after blood sampling, whereas glutamine levels were decreased longer before diagnosis (Fig. 2C and 2G). Histidine levels were lower in both fasting and nonfasting individuals (Fig. 2H), whereas the effect of fasting status on glutamine levels is difficult to ascertain given the differences between cohorts in fasting status (Fig. 2D). The branched chain amino acids, leucine, valine, and isoleucine, reported earlier by Mayers *et al.* (11), were not different between cases and controls (unadjusted *P* values of 0.75, 0.94, and 0.61, respectively).

**Table 2.** Top Hits From Logistic Regression Analysis

Metabolite^*a*^	Estimate^*b*^	SE	*z* Value	*P* Value	Adjusted *P* Value
Histidine	−0.188	0.074	−2.529	0.011	0.4274
Glutamine	−0.175	0.069	−2.525	0.012	0.4274
DHA.FA	0.195	0.081	2.393	0.017	0.5214
FAw3.FA	0.170	0.075	2.272	0.023	0.6203
M.HDL.P	−0.151	0.072	−2.085	0.037	0.7646
M.HDL.L	−0.149	0.072	−2.076	0.038	0.7695
DHA	0.153	0.075	2.029	0.043	0.7975
M.HDL.PL	−0.145	0.072	−2.020	0.043	0.8016
M.HDL.C	−0.139	0.071	−1.941	0.052	0.8513
M.HDL.CE	−0.138	0.072	−1.929	0.054	0.8589
M.HDL.PL	0.141	0.074	1.898	0.058	0.8756

Abbreviations: DHA, docosahexaenoic acid; HDL, high-density lipoprotein; DHA.FA, ratio of docosahexaenoic acid to all fatty acids; FAw3.FA, ratio of *ω*-3 fatty acids to total acids; M.HDL.P, concentration of medium HDL particles; M.HDL.PL: phospholipids in medium-sized HDL particles.

^a^ Logistic regression with single metabolite measure, sex, age, BMI, smoking status, T2DM status, fasting status, and cohort as covariates.

^b^ The estimates are the fitted *β* coefficients from the logistic regression model. As the input metabolite data were scaled, the estimates can be interpreted as follows: the OR for developing pancreatic cancer in a case with a typical low metabolite score of 1 SD below the average *z* score (= −1) would amount to 1.22 for *β* of −0.1 and 1.49 for *β* of −0.2. The *z* value mentioned in the table is the test statistic from the logistic regression models.

The results above were recapitulated in a minimally adjusted model, only corrected for sex and age (32). Glutamine and histidine were still among the top hits, with *P* values of 0.0063 and 0.00045 (not adjusted for multiple testing), respectively.

**Figure 3.**
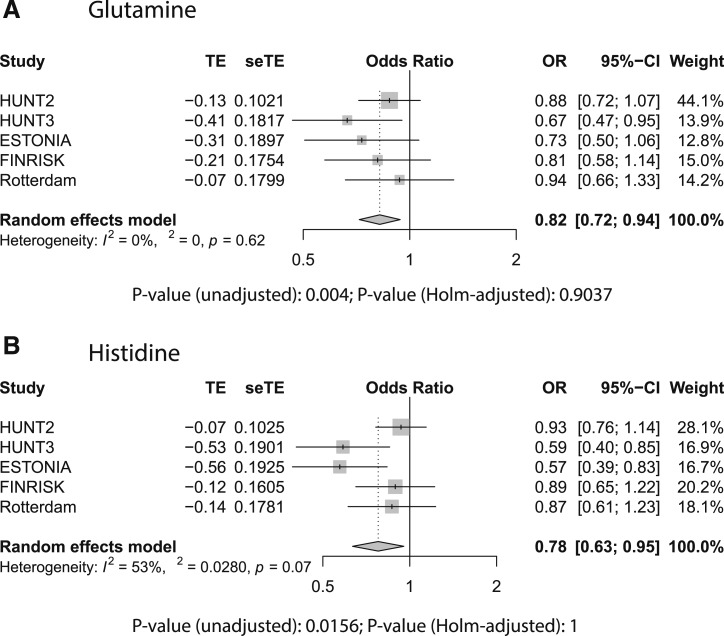
Forest plots from random effects meta-analysis across different cohorts for (A) glutamine and (B) histidine. The meta-analysis was performed on the *β* coefficients and SD from the logistic regressions run for each cohort separately. In the logistic regression, pancreatic cancer status was modeled as a function of log-transformed and standardized metabolite concentration, sex, age, BMI, smoking status, T2DM, and fasting status. Shown are the estimated effect size, the SE on this estimate, the estimated OR and the CI on this ratio, the weight of the individual cohort on the calculation of the final estimate, the heterogeneity measure (modeling differences between cohorts), and the unadjusted and Bonferroni–Holm-corrected *P* values for the respective metabolites.

To further address potential cohort differences, we performed a meta-analysis on the *β* coefficients from the logistic regression models that were applied per cohort. The results are summarized in Table 3 and provided in full in an online repository (32). The results from the meta-analysis corroborated our findings on the pooled data, with lower glutamine levels seen for all cohorts (unadjusted *P* value of 0.0040), but most prominently in HUNT3 (Fig. 3A), and lower histidine levels mostly in HUNT3 and EGCUT (unadjusted *P* value of 0.0022) (Fig. 3B, with similar trends in other cohorts and evidence for significant heterogeneity between cohorts). The mean ORs for an increase of 1 SD in glutamine or histidine levels were 0.82 and 0.78 (or 1.22 and 1.28 for a decrease of 1 SD), respectively. The meta-analysis provided some evidence for the involvement of *ω*-3 fatty acids (including docosahexaenoic acid and high-density lipoproteins).

**Table 3.** Top Hits From Meta-Analysis

Metabolite^*a*^	*β*	CI	Unadjusted *P* Value	*P* Value	*I* ^2^ (%)
Glutamine	−0.19538	−0.33:−0.06	0.004	0.9037087	0
DHA.FA	0.17259	0.04:0.3	0.0083	1	0
M.HDL.PL	−0.17905	−0.32:−0.04	0.0103	1	0
M.HDL.P	−0.17856	−0.32:−0.04	0.0104	1	0
M.HDL.L	−0.17732	−0.31:−0.04	0.0104	1	0
FAw3.FA	0.16222	0.03:0.29	0.0126	1	0
Histidine	−0.25164	−0.46:−0.05	0.0156	1	0.53
M.HDL.FC	−0.15636	−0.29:−0.02	0.0251	1	0
M.HDL.C	−0.15174	−0.29:−0.02	0.0267	1	0
M.HDL.CE	−0.14723	−0.28:−0.01	0.0306	1	0
DHA	0.13222	0:0.26	0.0438	1	0

Abbreviations: DHA, docosahexaenoic acid; DHA.FA, ratio of docosahexaenoic acid to total fatty acids; FAw3.FA, ratio of *ω*-3 fatty acids to total acids; HDL, high-density lipoprotein; M.HDL.C, total cholesterol in medium-sized HDL particles; M.HDL.CE, cholesterol esters in medium-sized HDL particles; M.HDL.FC, free cholesterol in medium-sized HDL particles; M.HDL.L, total lipids in medium-sized HDL particles; M.HDL.P, concentration of medium-sized HDL particles; M.HDL.PL, phospholipids in medium-sized HDL particles.

^a^ Meta-analysis across the five cohorts of logistic regression results with single metabolite measure, sex, age BMI, smoking status, T2DM status, and fasting status as covariates. *β* is effect size and can be interpreted as detailed in footnote *b* to Table 2. *P* value is Bonferroni–Holm-adjusted *P* value. *I*^2^ is the statistic used for heterogeneity between cohorts.

To provide a better understanding of the lower glutamine or histidine levels, we stratified the cohorts in quintiles based on the glutamine or histidine levels in controls. Individuals within the lowest 20% of glutamine levels ran a 1.5-fold elevated risk of pancreatic cancer, and individuals within the lowest 20% of histidine levels ran a 2.2-fold elevated risk of pancreatic cancer (Table 4).

**Table 4.** ORs for Developing Pancreatic Cancer in Different Glutamine and Histidine Strata

	Based on Control Data	Controls, n	Cases, n	OR	5% CI	95% CI	*P* Value
Glutamine							
0%	0.269	180	94	1	—	—	
20%	0.4538	176	66	0.72	0.49	1.05	0.0852
40%	0.487	177	62	0.67	0.46	0.98	0.0404
60%	0.5157	176	71	0.77	0.53	1.12	0.1734
80%	0.55358	178	62	0.66	0.46	0.98	0.0376
Histidine							
0%	0.03927	178	110	1	—	—	
20%	0.060498	177	71	0.65	0.45	0.93	0.0199
40%	0.064778	177	58	0.53	0.36	0.78	0.0011
60%	0.068174	177	66	0.6	0.42	0.87	0.0073
80%	0.072638	178	51	0.46	0.31	0.69	0.0001

### LASSO regression

**Figure 4.**
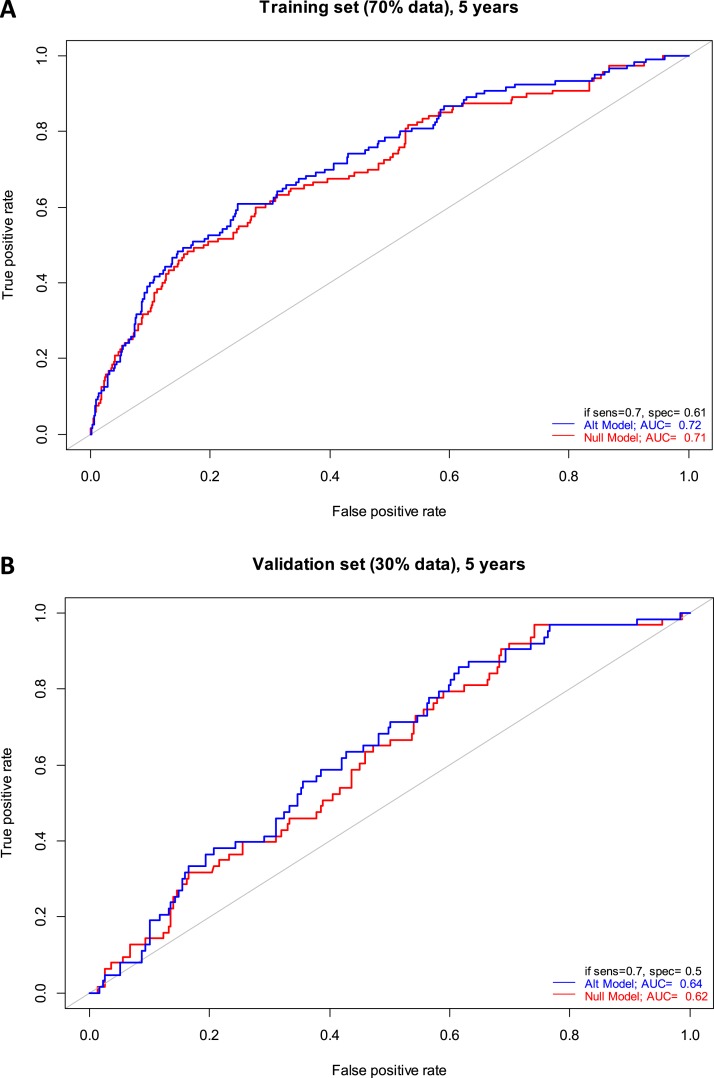
Receiver operator curves for classification of pancreatic cancer cases (sampled up to 5 y before diagnosis) and controls for (A) training set (70% of all individuals) and (B) performance testing set (30% of all individuals unseen during the variable selection). In red, the null model is shown in which only the clinical covariates (sex, age, BMI, smoking status, T2DM, and fasting status) were included in the regression. In blue, the alternative model is shown where the metabolites selected by the LASSO regression were included in addition to the clinical covariates. The AUCs are indicated, as well as the specificity (1 − false-positive rate) at 70% sensitivity.

LASSO regression was used to evaluate the additional predictive value of metabolomics biomarkers over clinical predictors. The performance of a reference (null) model, in which only the clinical covariates were used for prediction, was compared with an alternative model, in which metabolites selected by the LASSO regression were added to the model. The cases were stratified according to the time until diagnosis (up to 2 years, up to 5 years, and all cases without temporal constraint). In the model with cases up to 2 years until diagnosis, the LASSO regression selected medium very low-density lipoprotein (VLDL), total unsaturated fatty acids, and saturated fatty acids to be included in the model (Table 5), but it did not affect the performance on the 30% of the data that were unseen during the selection of the metabolites. In the model with cases up to 5 years until diagnosis, the LASSO regression model selected small VLDL and glutamine (consistent with the prominent decrease of glutamine levels in cases between 2 and 5 years before diagnosis) (Table 5). The performance of the alternative model increased slightly for both the training (AUC of 0.72 vs 0.71 for the null model, Fig. 4A) and the validation set (AUC of 0.64 vs 0.62 for the null model, Fig. 4B). In the model with all cases included, more metabolites were selected (Table 5), but the performance of the model including the metabolites on both training and validation set (AUC of 0.68 and 0.62, respectively) was worse than for the model with cases up to 5 years.

**Table 5.** Variables Selected by the LASSO Regression

Cohort	Time Condition	*λ*	Selected Variables	*P* Value	Significance
Full data	2 y	6.06	M.VLDL.FC_, UnSat, SFA.FA	0.175	
Full data	5 y	28.3	S.VLDL.FC_, Gln	0.0114	*P* < 0.05
Full data	Max(*t*)	2.02	XL.VLDL.TG, XL.HDL.TG, M.HDL.PL, XXL.VLDL.PL_., XXL.VLDL.CE_, L.VLDL.PL_, L.VLDL.FC_, M.LDL.TG_, XL.HDL.CE_, XL.HDL.FC_, L.HDL.FC_, FreeC, SMs, LA, DHA.FA, LA.FA, Glc, Cit, Ala, Gln, His, Val, Phe, AcAce, bOHBut, Crea	0.102	


The results of the cross-validated LASSO-penalized logistic regression for the full dataset are shown. For each regression the penalty parameter (*λ*) and the selected covariates (separated by commas) are given. For every model where metabolites were selected, the significance of the presence of all of the selected metabolites in the model, compared with the model without presence of metabolites, is tested in a global test, and its *P* value is given here. Note that the *P* value is only for the metabolites, not for the clinical covariates.

Abbreviations: AcAce, acetoacetate; Ala, alanine; bOHBut, 3-hydroxybutyrate; Cit, citrate; Crea, creatinine; DHA.FA, ratio of docosahexaenoic acid to total fatty acids; FreeC, free cholesterol; Glc, glucose; Gln, glutamine; His, histidine; LA, linoleic acid; LA.FA, ratio of linoleic acid to total fatty acids; L.HDL.FC_, free cholesterol to total lipids ratio in large HDLs; L.VLDL.FC_, free cholesterol to total lipids ratio in large VLDLs; L.VLDL.PL_, phospholipids to total lipids ratio in large VLDLs; M.HDL.PL, phospholipids in medium-sized HDLs; M.LDL.TG_, triglycerides to total lipids ratio in medium LDLs; M.VLDL.FC_, free cholesterol to total lipids ratio in medium VLDLs; Phe, phenylalanine; SFA.FA, ratio of saturated fatty acids to total fatty acids; SM, sphingomyelin; S.VLDL.FC_, free cholesterol to total lipids ratio in small VLDLs; UnSat, estimated degree of unsaturation; Val, valine; XL.HDL.CE_, cholesterol ester to total lipids ratio in very large HDLs; XL.HDL.FC_, free cholesterol to total lipids ratio in very large HDLs; XL.HDL.TG, triglycerides in very large HDLs; XXL.VLDL.CE_, cholesterol esters to total lipids ratio in chylomicrons and extremely large VLDLs; XL.VLDL.TG, triglycerides in extra-large VLDL particles; XXL.VLDL.PL_, phospholipids to total lipids ratio in chylomicrons and extremely large VLDLs.

## Discussion

Pancreatic cancer is usually diagnosed in an advanced stage of the disease, resulting in a poor prognosis. Most pancreatic cancer biomarker studies executed until today (9, 10, 13–16) collected samples at the time of diagnosis or even later, and therefore have limited clinical utility. However, they may provide insight in the pathophysiology of the disease. The setup of our study allowed for the identification of biomarkers in individuals who were not yet diagnosed with pancreatic cancer, and made efficient use of the large-scale biobanking infrastructure in Europe (BBMRI-LPC program).

We identified two potential biomarkers, glutamine and histidine, while noting that the differences between cases and controls were small and did not survive stringent multiple testing procedures, and that the clinical utility of these biomarkers is currently low. The increased risk of pancreatic cancer associated with low levels of glutamine and histidine was calculated to be only 1.5-fold to 2.2-fold and does not add much in terms of predictive potential to well-known risk factors for pancreatic cancer such as age, smoking, and T2DM. However, also earlier studies provided evidence for alterations in glutamine and histidine in pancreatic cancer (10, 15, 16, 33), suggesting that these may indeed be associated with pancreatic cancer–associated changes in metabolism. In the largest study by Fukutake *et al.* (15) (N = 360 vs 8372), histidine was found particularly low in patients with resectable disease stage 0-IIB. This group of patients in a relatively early state of the disease is likely most similar to our group of individuals who were diagnosed in <2 years after blood sampling and had the lowest histidine levels of all cases. Also, in other cancer-related studies, negative correlations between histidine levels and cancer incidence and/or cancer-associated mortality were observed (34–36). Remarkably, a recent report demonstrated also lower efficacy of cancer treatment in individuals with low histidine levels, and suggested histidine supplementation to enhance the efficacy of methotrexate treatment in leukemia (37). In a study by Roux *et al.* (33), human pancreatic ductal adenocarcinoma (PDAC) cell lines displayed higher glutamine uptake and metabolism than did non-PDAC cancer cell lines, in line with our study. Moreover, mouse models in which human PDAC cells were injected into the pancreas demonstrated lower levels of circulating glutamine than control animals, which could not be explained by inflammation of the pancreas nor by the development of T2DM (33). This makes it unlikely that the identification of glutamine and histidine in our study is due to pancreatitis, often associated with pancreatic cancer, but we can only formally exclude this possibility by including a cohort of patients with chronic inflammation of the pancreas.

One of the reasons why changes in metabolites such as glutamine and histidine are difficult to detect is that the concentrations of these metabolites are relatively high, and that local events, such as a pancreatic tumor, contribute only little to the overall pool of these metabolites. Other metabolites may be more specific to the metabolism in the pancreas and may show more prominent changes. These types of metabolites require broader metabolomic screening than the Nightingale platform provides. Although having superior robustness and throughput and low cost, the range of metabolites measured on the Nightingale platform is limited to amino acids, other polar metabolites, and a large range of lipid and lipoprotein classes. Our study calls for the use of complementary biomarker platforms on these samples, and suggests to limit the sampling to within 5 years before diagnosis and not beyond.

Our study was not able to replicate the findings from the single study with a design and sample size comparable to ours (11). This study identified the branched-chain amino acids valine, leucine, and isoleucine as potential prognostic biomarkers for pancreatic cancer. We did not find any difference between cases and controls for these amino acids, nor were our top metabolites identified in this earlier study. This may be a reflection of the limited power of both studies for the discovery of small changes observed for these metabolites. However, we did not even observe trends in the same directions. Differences in the measurement platforms (^1^H-NMR vs liquid chromatography followed by mass spectrometry) may play a role, but the different amino acids can robustly be measured by both. It is equally plausible that the differences are due to differences in the studied populations or confounding factors, which were not or were incompletely corrected for in the statistical model, such as nutrition.

In conclusion, our study lends initial support to the existence of metabolic alterations in early pancreatic cancer development, highlighting glutamine and histidine as metabolites of interest, but also underscores the challenges to find robust, prognostic biomarkers for rare disorders. To address this, larger studies are needed, including more metabolites with lower concentrations and/or integrated studies at multiple “omics” levels.

## Data Availability

Restrictions apply to the availability of data generated or analyzed during this study to preserve patient confidentiality or because they were used under license. The corresponding author will on request detail the restrictions and any conditions under which access to some data may be provided.

## Abbreviations:

^1^H-NMR: proton nuclear magnetic resonance
AUC: area under the receiver operator curve
BBMRI-LPC: Biobanking and Biomolecular Resources Research Infrastructure Large Population Cohorts
BMI: body mass index
ECGUT: Estonian Genome Center of the University of Tartu study
FR: FINRISK Study
HUNT: Nord-Trøndelag Health Study
LASSO: least absolute shrinkage and selection operator
PDAC: pancreatic ductal adenocarcinoma
RS: Rotterdam Study
T2DM: type 2 diabetes mellitus
VLDL: very low-density lipoprotein
